# Low serum dehydroepiandrosterone sulfate is related to diabetic retinopathy in males with type 2 diabetes mellitus

**DOI:** 10.1186/s12967-026-08121-1

**Published:** 2026-04-16

**Authors:** Shouxia Li, Chaoyu Zhu, Fusong Jiang, Yuanyuan Xiao, Qianqian Wang, Wenjing Song, Qingge Gao, Xinyi Wang, Li Wei

**Affiliations:** Department of Endocrinology, Shanghai Sixth People’s Hospital, Shanghai Jiao Tong University School of Medicine, Shanghai, China

**Keywords:** Androgen, Type 2 diabetic retinopathy, Dehydroepiandrosterone sulfate, Androstenedione, Total testosterone

## Abstract

**Objective:**

Androgens are increasingly recognized as important regulators of metabolic balance and vascular integrity. However, their relationship with diabetic retinopathy (DR) remains incompletely defined and appears to differ by sex. The present study aimed to examine the associations between circulating levels of total testosterone (TT), dehydroepiandrosterone sulfate (DHEAS), and androstenedione (ASD) and DR risk among individuals with type 2 diabetes mellitus (T2DM), with particular emphasis on gender-stratified analyses.

**Methods:**

This cross-sectional investigation analyzed 796 hospitalized T2DM cases. Serum concentrations of TT, DHEAS, and ASD were quantified using chemiluminescence immunoassays. Multivariable logistic regression was utilized to assess the associations between androgen levels and the presence of DR, with analyses conducted separately for men and women to account for sex-specific differences.

**Results:**

Among male participants, after comprehensive adjustment for potential confounding variables, serum DHEAS levels were independently and inversely linked with DR risk (tertile 3 vs. tertile 1: OR = 0.43, 95% CI: 0.23–0.82; P for trend = 0.007). Restricted cubic spline modeling demonstrated an approximately linear inverse dose-response relationship between DHEAS concentrations and DR risk (overall *P* < 0.001; nonlinear *P* = 0.193). No correlations were found between serum TT or ASD levels and DR in men. In female patients, an inverse relationship between TT and DR was attenuated and rendered nonsignificant after full multivariable adjustment, and neither DHEAS nor ASD showed a statistically significant association with DR risk.

**Conclusions:**

Lower serum DHEAS concentrations are independently linked with elevated DR risk in males with T2DM, suggesting a potential role for DHEAS in the pathophysiology of DR in males.

## Introduction

Diabetic retinopathy (DR) is a frequent and defining microvascular complication of the disease and remains the most common reason for irreversible vision loss among adults globally, creating a substantial clinical and socioeconomic burden [1, 2]. Although established risk factors like chronic hyperglycemia, hypertension, and diabetes duration are known to contribute to DR development, considerable variability exists in disease onset and progression among individuals, indicating that additional, less well-characterized mechanisms are involved [3, 4]. Recently, increasing attention has been directed toward the role of sex hormones in metabolic regulation and vascular health. Androgens, including total testosterone (TT) and its adrenal precursors dehydroepiandrosterone sulfate (DHEAS), dehydroepiandrosterone (DHEA), and androstenedione (ASD), extend far beyond their traditional roles in reproductive physiology. These hormones influence endothelial function, inflammatory signaling, oxidative stress responses, and neuronal survival [5–7]. Importantly, DR is now recognized as a neurovascular inflammatory disorder characterized by disruption of the blood-retinal barrier, persistent low-grade inflammation, and progressive retinal neurodegeneration [8, 9]. Experimental studies have shown that several androgens possess anti-inflammatory, antioxidant, and neuroprotective properties, closely aligning with the core pathological processes underlying DR [10–12].

Despite these mechanistic insights, clinical evidence linking androgens to DR remains limited and inconclusive. A population-based investigation from the Netherlands observed no marked associations between circulating DHEA levels and markers of retinal microvascular damage [13]. In contrast, a study on T2DM in China reported that reduced DHEA was linked to a greater likelihood of diabetic microvascular complications [14]. With respect to TT, previous research has demonstrated correlations between TT concentrations and metabolic parameters such as adiposity and glycemic control, suggesting that metabolic syndrome-related alterations in androgen levels may indirectly influence DR risk [15]. Additionally, another study identified a dose-dependent relationship between TT fluctuations and DR risk in men, while also emphasizing that higher TT levels are not uniformly protective [16]. Although previous studies have provided valuable insights into the relationship between DHEA/DHEAS and DR [13, 14], the specific associations of DHEAS and ASD—two major circulating androgens with distinct biological properties—with DR risk remain incompletely characterized. DHEAS, the sulfated ester of DHEA, is the most abundant circulating androgen in humans and exhibits greater stability and a longer half-life than DHEA, making it a more reliable biomarker for epidemiological studies [17]. ASD, as a direct precursor of both testosterone and estrone, occupies a central position in steroidogenesis and may reflect adrenal and gonadal androgen synthesis capacity [18]. Despite the significant biological importance of these two hormones, research systematically evaluating their associations with DR remains relatively limited. Furthermore, existing studies typically focus on a single hormone in isolation, which precludes direct comparisons of the differences in association strength between the two hormones within the same study population.

This study employed a cross-sectional approach to simultaneously quantify TT, DHEAS, and ASD using standardized chemiluminescence immunoassays. Through rigorous multivariate adjustments for various confounding factors and an emphasis on gender-stratified analysis to reveal potential differential effects. The methodology allows us to determine whether the observed associations are hormone-specific or reflect broader alterations in androgen metabolism, thereby providing novel insights into the hormonal mechanisms underlying the pathophysiology of DR.

## Materials and methods

### Study subjects

This cross-sectional investigation consecutively enrolled individuals with T2DM who had been hospitalized in the Department of Endocrinology and Metabolism at Shanghai Sixth People’s Hospital (Lin-gang Branch) between January 1, 2023, and October 31, 2025. Exclusion criteria included best-corrected visual acuity below 0.3 (this threshold was selected based on WHO criteria to ensure reliable fundus photography and DR grading, as lower acuity may compromise image quality or reflect ocular pathologies that could confound retinopathy assessment. Patients with prior ocular interventions were also excluded to ensure the study population was suitable for accurate DR evaluation); a history of intraocular surgery, glaucoma, retinal laser treatment, or retinal detachment; acute cardiovascular or cerebrovascular events; chronic kidney disease stage 4 or higher; malignancy; use of diabetic hormone therapy; pregnancy; and incomplete fundus photography data. The study protocol received approval from the Ethics Committee of Shanghai Sixth People’s Hospital (Approval No.: 2023-KY-139(K)). Informed consent was waived due to the use of anonymized electronic medical records.

### Data collection

Demographic characteristics, including age and sex, as well as behaviors such as smoking and alcohol consumption, were obtained from electronic medical records. Medical history data encompassed hypertension (HTN), coronary heart disease (CHD), and stroke. Anthropometric measurements were collected to calculate body mass index (BMI) as weight (kg) divided by the square of the height (m²). Laboratory assessments included fasting blood glucose (FBG), total cholesterol (TC), triglycerides (TG), high-/low-density lipoprotein cholesterol (HDL-C/LDL-C), 2-hour postprandial glucose (2 h-PPG), hemoglobin A1c (HbA1c), glycated albumin (GA), fasting insulin (FINS), 2-hour postprandial insulin (2 h-PI), fasting C-peptide (FCP), 2-hour postprandial C-peptide (2 h-PCP), serum uric acid (SUA), urine albumin-to-creatinine ratio (UACR), serum creatinine (Cr), and 24-hour urinary microalbumin (24 h-UM). The estimated glomerular filtration rate (eGFR) was determined using the Chronic Kidney Disease Epidemiology Collaboration equation [19].

### Androgen measurements

Samples of fasting venous blood were obtained the morning after admission and were processed immediately in the clinical laboratories of Shanghai Sixth People’s Hospital. Serum concentrations of TT, DHEAS, and ASD were measured using chemiluminescence immunoassays. After appropriate sample preparation, analyses were performed on the LIAISON^®^ fully automated chemiluminescence immunoassay platform.

### Definitions

Diabetes was defined based on standard criteria: FBG ≥ 7.0 mmol/L, 2 h-PPG ≥ 11.1 mmol/L, HbA1c ≥ 6.5%, self-reported disease, or current treatment with glucose-lowering medications [20]. Hypertension was identified based on self-reported hypertension, systolic blood pressure ≥ 140 mmHg, diastolic blood pressure ≥ 90 mmHg, or treatment with antihypertensive drugs [21]. CHD was identified based on self-reports of myocardial infarction, angina, or coronary artery bypass graft surgery. Stroke status was determined by self-report, including both ischemic and hemorrhagic stroke events.

### Diagnosis of diabetic retinopathy

DR was assessed by experienced ophthalmologists using standardized non-mydriatic fundus photography. Disease severity was graded into five categories according to the International Clinical Diabetic Retinopathy Disease Severity Scale (Table 1) [22]. For analytical purposes in this cross-sectional study, participants classified as grade 0 were assigned to the Non-DR group, whereas those classified as grades 1 through 4 were grouped as having DR.

**Table 1:** International clinical classification of diabetic retinopathy (2025 edition)

Grade	Disease severity	Findings of dilated fundus examination
0	No apparent retinopathy	No abnormalities
1	Mild NPDR	Only microaneurysms are present
2	Moderate NPDR	Progression of microvascular damage, presenting with retinal hemorrhages, hard exudates, cotton wool spots, and other lesions
3	Severe NPDR	Meet any of the following criteria: (1) Retinal hemorrhages in 4 quadrants; (2) Venous beading in 2 quadrants; (3) Significant intraretinal microvascular abnormalities in 1 quadrant
4	Very severe NPDR	Extensive retinal hemorrhages/microaneurysms with venous tortuosity

NPDR: Non-Proliferative Diabetic Retinopathy

### Statistical analyses

Continuous variables are given as means ± standard deviation when normally distributed, as confirmed by normality testing, and were compared using independent-samples t-tests. Skewed variables are medians (interquartile range) and compared using Mann-Whitney U tests. Categorical data are given as counts and percentages and were assessed using chi-square tests. To evaluate the links between circulating androgens and DR, multivariable binary logistic regression analyses were performed. Androgen concentrations were classified into tertiles, with the lowest (tertile 1) serving as the reference. Three hierarchical adjustment models were constructed: Model 1 included adjustment for age; Model 2 had further adjustments for smoking and alcohol consumption; and Model 3 was additionally adjusted for diabetes duration, BMI, HTN, CHD, FBG, 2 h-PPG, FINS, 2 h-PI, FCP, 2 h-PCP, GA, SUA, eGFR, UACR, and 24 h-UM. In supplementary analyses, androgen levels were also examined as continuous variables following natural logarithmic transformation and shown as increments in the standard deviation. The restricted cubic spline (RCS) analysis method was utilized, incorporating the 10th, 50th, and 90th percentiles as knot positions, to investigate the potential dose-response relationship between serum DHEAS concentration and the risk of DR among male participants, as specified in Model 3. All statistical analyses were conducted using SPSS 27.0 and R 4.1.3, with a two-sided *P* < 0.05 considered significant.

## Results

### Participant characteristics

Seven hundred and ninety-six individuals with T2DM were enrolled. Of these, 520 were men (65.3%) and 276 were women (34.7%). Among male participants, the median age at DR diagnosis was 58 years, and the median duration of diabetes was 8 years. Female participants with DR had a higher median age (62 years) and a longer median disease duration (11 years). Detailed demographic, clinical, and biochemical characteristics stratified by sex and DR status are presented in Table 2. In men, those with DR were markedly older and had longer disease durations than those without DR. Additionally, male patients with DR exhibited higher SUA, UACR, and 24 h-UM levels, along with significantly lower FBG, fasting and 2-h postprandial insulin concentrations, and fasting and 2 h-PCP levels (all *P* < 0.05). Importantly, serum DHEAS concentrations were substantially reduced in men with DR compared with their non-DR counterparts (*P* < 0.05), whereas TT and ASD showed no marked changes. Similarly, among female participants, DR was associated with older age and longer diabetes duration, as well as elevated UACR and 24 h-UM levels. Women with DR also demonstrated lower fasting and 2 h-PI 2 h-PCP levels, reduced eGFR, and higher levels of 2 h-PPG, GA, and Cr (all *P* < 0.05). The TT concentrations in the sera were markedly decreased in women with DR compared with those without DR (*P* < 0.05), while no differences were found for DHEAS or ASD.

**Table 2:** Demographic characteristics and clinical parameters of DR and Non-DR patients

	Men (*n* = 520)			Women (*n* = 276)		
	Non-DR	DR	*P*	Non-DR	DR	*P*
Participants, %	291(56.0)	229(44.0)	-	168(60.9)	108(39.1)	-
Age, years	53.00(45.00, 60.00)	58.00(47.00, 64.00)	**< 0.001**	58.00(44.00, 64.00)	62.00(54.00, 66.00)	**< 0.001**
Duration of type 2 diabetes, years	7.00(2.00,10.00)	8.00(4.00, 15.00)	**0.002**	6.00(1.00,13.00)	11.00(6.00, 18.00)	**< 0.001**
BMI, Kg/m^2^	25.98 ± 3.72	25.94 ± 3.23	0.692	26.23 ± 3.35	25.68 ± 3.28	0.186
HTN, %	124(42.6)	130(56.8)	0.089	91(54.2)	69(63.9)	0.110
CHD, %	21(7.2)	27(11.8)	0.799	10(6.0)	12(11.1)	0.123
Stroke, %	21(7.2)	18(7.9)	0.270	7(4.2)	10(9.3)	0.086
Current smoking, %	109(37.5)	60(26.2)	0.064	11(6.5)	9(8.3)	0.577
Current drinking, %	39(13.4)	26(11.4)	0.483	20(11.9)	12(11.1)	0.841
TC, mmol/L	4.70 ± 1.60	4.73 ± 1.54	0.711	4.77 ± 1.15	5.00 ± 1.40	0.133
TG, mmol/L	1.65(1.00, 2.91)	1.64(0.99, 2.65)	0.643	1.52(1.10, 2.30)	1.59(0.98, 2.25)	0.781
HDL-C, mmol/L	1.12 ± 0.30	1.10 ± 0.29	0.500	1.23 ± 0.43	1.23 ± 0.25	0.990
LDL-C, mmol/L	2.97 ± 1.04	3.03 ± 0.98	0.555	3.11 ± 0.89	3.18 ± 0.95	0.513
FBG, mmol/L	7.03(5.79, 10.26)	6.82(5.43, 8.50)	**0.030**	7.25(5.76, 9.47)	7.47(5.71, 9.72)	0.744
2 h-PPG, mmol/L	12.15(8.37, 16.36)	11.33(8.23, 14.82)	0.086	11.91(8.99, 15.54)	13.93(10.16, 17.16)	**0.026**
FINS, uU/mL	5.09(3.08, 10.89)	3.82(2.18, 6.66)	**< 0.001**	6.21(3.82, 11.67)	4.93(2.88, 11.25)	**0.021**
FCP, ng/mL	1.56(1.09, 2.40)	1.36(0.90, 1.99)	**0.010**	1.82(1.23, 2.38)	1.41(0.94, 2.18)	**0.002**
HbA1c, %	8.84 ± 2.01	9.12 ± 1.93	0.176	8.59 ± 1.98	8.79 ± 1.89	0.414
GA, %	23.66 ± 6.93	24.61 ± 6.93	0.157	22.39 ± 6.55	24.62 ± 5.88	**0.004**
2 h-PI, uU/mL	20.94(13.35, 40.19)	17.34(10.25, 33.53)	**0.009**	26.80(16.35, 46.02)	20.20(10.81, 33.91)	**< 0.001**
2 h-PCP, ng/mL	3.94(2.70, 5.75)	3.27(2.19, 5.30)	**< 0.001**	4.09(2.82, 6.62)	3.62(2.20, 5.07)	**< 0.001**
Cr, µmol/L	71.11 ± 13.82	68.72 ± 13.81	0.069	52.31 ± 11.44	56.66 ± 14.39	**0.006**
SUA, µmol/L	351.08 ± 88.76	384.73 ± 85.18	**< 0.001**	292.86 ± 73.50	300.00 ± 69.37	0.415
eGFR, mL/(min*1.73 m^2^)	119.53 ± 33.37	120.04 ± 34.97	0.837	138.92 ± 40.05	124.72 ± 43.21	**0.006**
UACR, µg/mg	12.13(4.63, 28.37)	19.46(9.50, 55.70)	**< 0.001**	8.11(5.65, 13.04)	13.40(7.50, 55.27)	**< 0.001**
24 h-UM, mg/24 h	11.63(6.97, 44.93)	21.56(8.40, 97.06)	**< 0.001**	8.42(5.97, 13.70)	10.68(6.44, 22.47)	**0.025**
TT, nmol/L	11.02(8.63, 14.70)	10.92(9.11, 14.62)	0.671	1.04(0.70, 1.59)	0.81(0.49, 1.19)	**< 0.001**
ASD, ng/mL	1.25(0.82, 1.82)	1.16(0.78, 1.61)	0.078	0.81(0.49, 1.19)	0.78(0.49, 0.98)	0.128
DHEAS, µg/dL	245.0(212.4, 259.7)	211.5(166.6, 249.9)	**< 0.001**	101.7(69.2, 128.2)	92.2(69.7, 133.4)	0.557

BMI, body mass index; HTN, Hypertension; CHD, coronary heart disease; TC, total cholesterol; TG, triglyceride; HDL-C, high-density lipoprotein cholesterol; LDL-C, low-density lipoprotein cholesterol; FBG, fasting blood glucose; 2 h-PPG, 2-hour postprandial blood glucose; HbA1c, hemoglobin A1c; GA, glycated albumin; FINS, fasting insulin; 2 h-PI, 2-hour postprandial insulin; FCP, fasting C-peptide; 2 h-PCP, 2-hour postprandial C-peptide; SUA, serum uric acid; UACR, urine albumin-to-creatinine ratio; Cr, creatinine; 24 h-UM, 24-hour urinary microalbumin; eGFR, estimated glomerular filtration rate; TT, total testosterone; DHEAS, dehydroepiandrosterone sulfate; ASD, androstenedione

### Relationships between hormone levels and DR prevalence

**Fig. 1:**
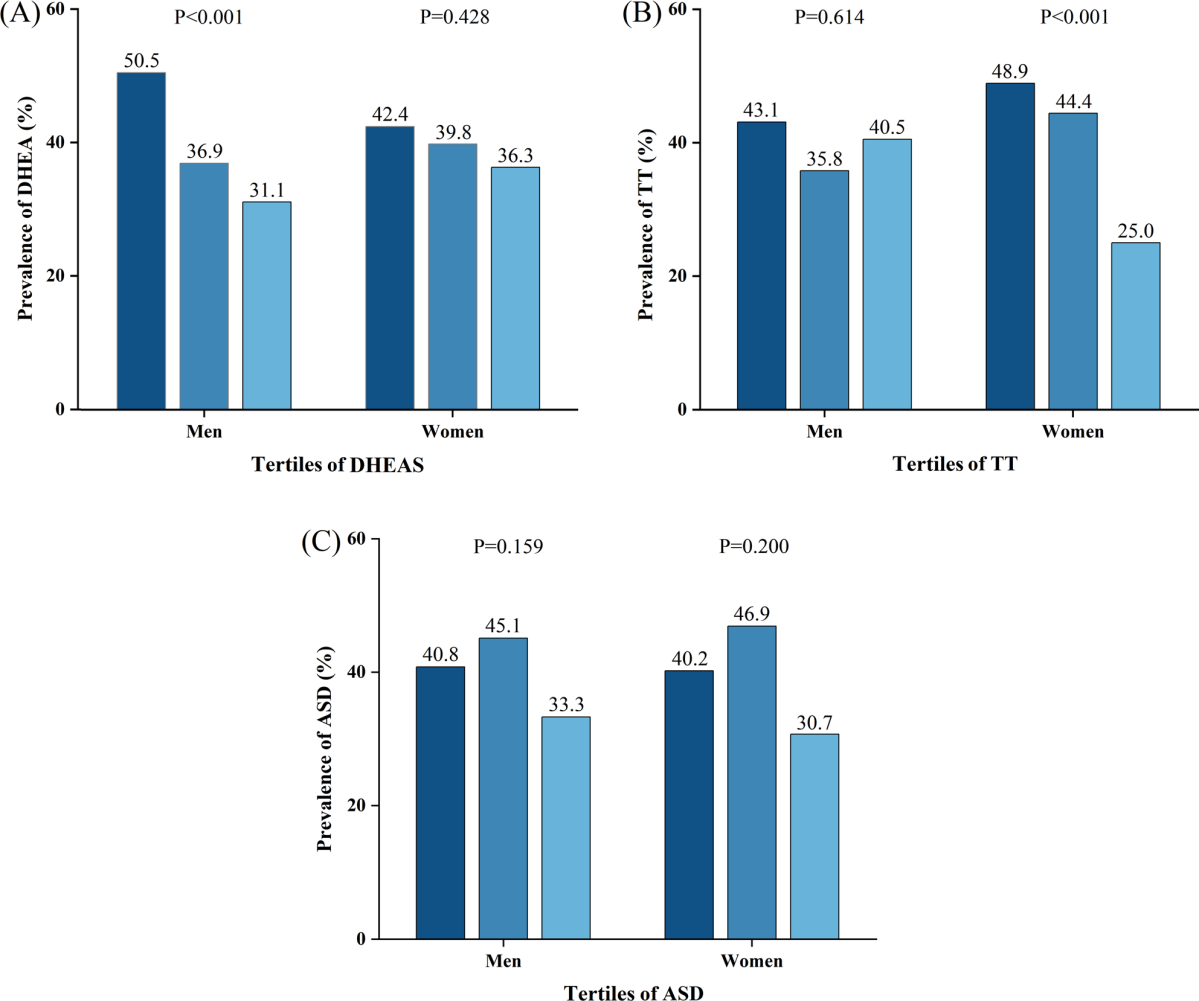
Incidence of DR by gender across tertiles of DHEAS (**A**), TT (**B**), and ASD (**C**)

Figure 1 illustrates the prevalence of DR across tertiles of androgen levels in males and females with T2DM. In men, the prevalence of DR declined progressively with increasing DHEAS levels (tertile 1: 50.5%; tertile 2: 36.9%; tertile 3: 31.1%; *P* < 0.001). No marked trends between DR prevalence and serum TT (tertile 1: 43.1%; tertile 2: 35.8%; tertile 3: 40.5%; *P*=0.641) or ASD (tertile 1: 40.8%; tertile 2: 45.1%; tertile 3: 33.3%; P༝0.159) levels were seen in male patients. In contrast, among women, a decreasing trend in DR prevalence was observed with increasing TT concentrations (tertile 1: 48.9%; tertile 2: 44.4%; tertile 3: 25.0%; *P* < 0.05). However, there were no marked links between DR prevalence and serum DHEAS (tertile 1: 42.4%; tertile 2: 39.8%; tertile 3: 36.3%; P༝0.428) or ASD (tertile 1: 40.2%; tertile 2: 46.9%; tertile 3: 30.7%; P༝0.200) levels in female participants.

### Association between androgen levels and DR risk

The odds ratios (ORs) for DR derived from multivariable logistic regression analyses are summarized in Tables 3 and 4 for men and women, respectively. In male patients, after full adjustment for potential confounders (Model 3), higher serum DHEAS levels were independently related to a lower risk of DR. Compared with tertile 1, both tertile 2 (OR = 0.41, 95% CI: 0.24–0.70) and tertile 3 (OR = 0.43, 95% CI: 0.23–0.82) exhibited significantly reduced odds of DR (P for trend < 0.05). When analyzed as a continuous variable following natural logarithmic transformation, each standard deviation rise in DHEAS was linked to a 42% decrease in DR risk (OR = 0.57, 95% CI: 0.42–0.71; *P* < 0.001). However, neither TT nor ASD were independently associated with DR risk in men. In females, serum TT demonstrated an inverse trend with DR risk in Models 1 and 2 (P for trend < 0.05). However, this association was not retained in Model 3 after full adjustments. Women showed no marked links between DHEAS or ASD levels and DR risk.

**Table 3:** Odds ratios of DR among men by different status of DHEAS, TT and ASD

	OR (95% CI)		
	Model 1	Model 2	Model 3
DHEAS (nmol/L)			
Tertile 1	Reference	Reference	Reference
Tertile 2	**0.39 (0.25–0.61)**	**0.40 (0.26–0.62)**	**0.42 (0.24–0.71)**
Tertile 3	**0.33 (0.20–0.53)**	**0.33 (0.20–0.55)**	**0.43 (0.23–0.82)**
P for trend	**< 0.001**	**< 0.001**	**0.007**
Per SD increment	**0.55 (0.44–0.67)**	**0.55 (0.45–0.68)**	**0.57 (0.42–0.71)**
TT (nmol/L)			
Tertile 1	Reference	Reference	Reference
Tertile 2	0.77 (0.50–1.19)	0.79 (0.51–1.23)	0.80 (0.47–1.38)
Tertile 3	1.00 (0.65–1.55)	0.98 (0.63–1.52)	0.96 (0.56–1.66)
P for trend	0.993	0.913	0.927
Per SD increment	1.04 (0.87–1.24)	1.04 (0.87–1.24)	1.08 (0.87–1.35)
ASD (nmol/L)			
Tertile 1	Reference	Reference	Reference
Tertile 2	1.34 (0.87–2.08)	1.39 (0.90–2.17)	2.18 (1.29–3.69)
Tertile 3	0.81 (0.52–1.27)	0.85 (0.54–1.34)	1.02 (0.60–1.75)
P for trend	0.371	0.462	0.953
Per SD increment	0.88 (0.73–1.06)	0.89 (0.73–1.08)	0.98 (0.78–1.24)

Model 1: adjusts for age

Model 2: model 1 + current smoking, current drinking

Model 3: model 2 + Duration of type 2 diabetes, BMI, HTN, CHD, FBG, 2 h-PPG, FINS, 2 h-PI, FCP, 2 h-PCP, GA, SUA、eGFR、UACR, and 24 h-UM

BMI, body mass index; HTN, Hypertension; CHD, coronary heart disease; FBG, fasting blood glucose; 2 h-PPG, 2-hour postprandial blood glucose; GA, glycated albumin; FINS, fasting insulin; 2 h-PI, 2-hour postprandial insulin; FCP, fasting C-peptide; 2 h-PCP, 2-hour postprandial C-peptide; SUA, serum uric acid; UACR, urine albumin-to-creatinine ratio; 24 h-UM, 24-hour urinary microalbumin; eGFR, estimated glomerular filtration rate

**Table 4:** Odds ratios of DR among women by different status of DHEAS, TT and ASD

	OR (95% CI)		
	Model 1	Model 2	Model 3
DHEAS (nmol/L)			
Tertile 1	Reference	Reference	Reference
Tertile 2	0.88 (0.48–1.61)	0.91(0.49–1.68)	0.93 (0.46–1.87)
Tertile 3	0.80 (0.43–1.47)	0.80 (0.43–1.47)	0.95 (0.47–1.93)
P for trend	0.466	0.466	0.879
Per SD increment	0.92 (0.71–1.18)	0.92 (0.71–1.19)	0.98 (0.72–1.34)
TT (nmol/L)			
Tertile 1	Reference	Reference	Reference
Tertile 2	0.80 (0.44–1.45)	0.79 (0.44–1.44)	0.66 (0.33–1.34)
Tertile 3	0.46(0.24–0.89)	0.44 (0.22–0.86)	0.46 (0.20–1.06)
P for trend	**0.024**	**0.018**	0.061
Per SD increment	0.66 (0.43–1.03)	0.65 (0.41–1.02)	0.68 (0.41–1.14)
ASD (nmol/L)			
Tertile 1	Reference	Reference	Reference
Tertile 2	1.27 (0.70–2.29)	1.27 (0.70–2.30)	0.80 (0.39–1.66)
Tertile 3	1.01 (0.52–1.96)	0.98 (0.50–1.92)	0.66 (0.29–1.48)
P for trend	0.900	0.969	0.302
Per SD increment	0.87 (0.65–1.15)	0.85 (0.64–1.13)	0.80 (0.57–1.14)

Model 1: adjusts for age

Model 2: model 1 + current smoking, current drinking

Model 3: model 2 + Duration of type 2 diabetes, BMI, HTN, CHD, FBG, 2 h-PPG, FINS, 2 h-PI, FCP, 2 h-PCP, GA, SUA、eGFR、UACR, and 24 h-UM

BMI, body mass index; HTN, Hypertension; CHD, coronary heart disease; FBG, fasting blood glucose; 2 h-PPG, 2-hour postprandial blood glucose; GA, glycated albumin; FINS, fasting insulin; 2 h-PI, 2-hour postprandial insulin; FCP, fasting C-peptide; 2 h-PCP, 2-hour postprandial C-peptide; SUA, serum uric acid; UACR, urine albumin-to-creatinine ratio; 24 h-UM, 24-hour urinary microalbumin; eGFR, estimated glomerular filtration rate

**Fig. 2:**
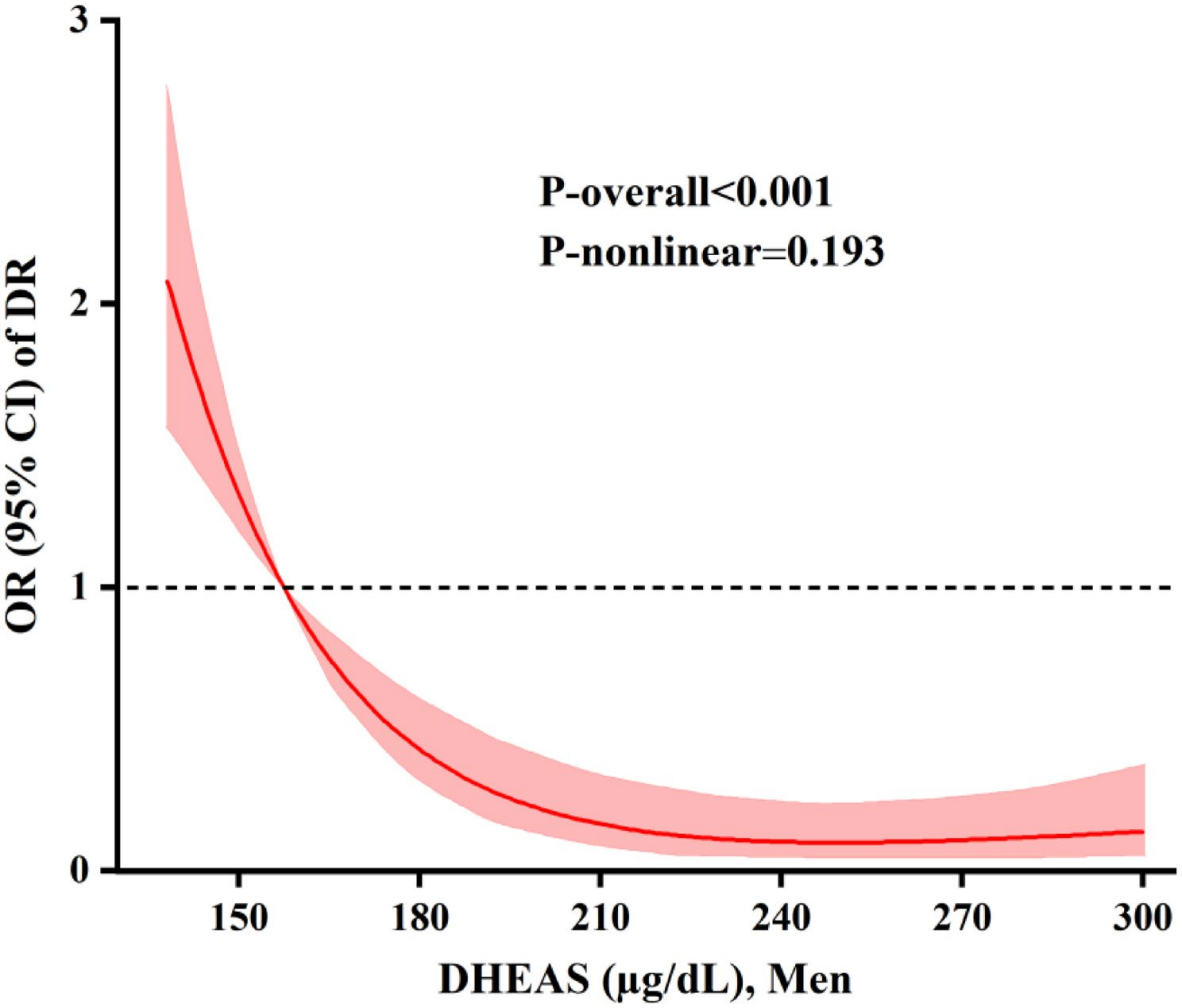
The dose-response relationship between DHEAS and DR in males

RCS analyses further highlighted an approximately linear inverse dose-response relationship between serum DHEAS concentrations and DR risk in male patients after multivariable adjustment (overall *P* < 0.001; P for nonlinearity = 0.193) (Fig. 2). Increasing DHEAS levels were consistently linked with progressively lower likelihood of DR.

## Discussion

This cross-sectional investigation provided a comprehensive examination of the links between circulating androgen concentrations including TT, DHEAS, and ASD, and the presence of DR in individuals with T2DM. By conducting sex-stratified analyses, we identified distinct gender-specific patterns. The principal finding of this study is that, among male patients, lower serum DHEAS concentrations were independently linked with a higher DR risk in a dose-dependent manner. However, no links were seen between androgens and DR in female patients after full adjustment for confounding factors. These findings underscore the likelihood that androgens exert sex-dependent effects in DR pathogenesis, suggesting that DHEAS may protect against DR specifically in men.

From a clinical epidemiology perspective, the sex-specific patterns observed in this study are consistent with the established differences in the prevalence of DR between men and women with type 2 diabetes. Large-scale epidemiological studies have consistently shown that men are at a higher risk of developing DR compared to women, particularly within middle-aged populations [23]. Previous research findings have demonstrated that male sex serves as an independent risk factor for the progression of retinopathy [24, 25]. This sex disparity may be partially explained by differences in androgen profiles. Men typically have circulating androgen levels that are significantly higher than those of women. These hormones are known to modulate endothelial function, inflammatory responses, and oxidative stress—all of which are key pathways in the pathogenesis of DR. Our findings indicate that low levels of DHEAS are independently associated with an increased risk of DR in male patients, while no such association was observed in females. This suggests that androgens may play a more significant protective role in the male retinal microvascular system. In contrast, the lack of this association in females may indicate the less dominant effect of androgen activity. Furthermore, the hormonal milieu in women experiences significant changes throughout their lifespan, particularly during menopause, when androgen levels decline in relation to estrogens. This decline may alter susceptibility to microvascular complications. These observations underscore the importance of incorporating sex-stratified approaches in both clinical risk assessment and future interventional studies targeting hormonal pathways in DR. From a clinical practice standpoint, measuring DHEAS levels in male patients with T2DM may assist in identifying individuals at an elevated risk for DR, beyond traditional factors such as glycemic control and diabetes duration. This could enable the development of more personalized surveillance strategies.

Accumulating evidence has highlighted a close relationship between DHEAS and macrovascular disease. For example, individuals with subclinical hypothyroidism have been reported to exhibit significantly reduced DHEAS concentrations compared with healthy controls, along with an inverse association between DHEAS levels and carotid intima-media thickness (CIMT) [26]. This observation implies that diminished DHEAS may contribute to arterial wall thickening and accelerate atherosclerotic processes in subclinical disease states. Consistently, data from the Rotterdam Study demonstrated a negative correlation between serum DHEAS levels and coronary artery calcification in men, suggesting a potential role for DHEAS in attenuating vascular calcification and macrovascular burden [13]. Moreover, studies conducted in diabetic populations have found lower DHEAS levels in male patients to be substantially related to elevated coronary heart disease risk, further supporting the importance of DHEAS in modulating cardiovascular and metabolic risk profiles [27]. The vascular effects of DHEAS are thought to involve multiple biological pathways, including the regulation of endothelial function, smooth muscle cell proliferation, inflammation, and vasomotor tone [28, 29].

In addition to its association with macrovascular disease, DHEAS has also been implicated in diabetic microvascular complications. A study involving male patients with T2DM across different age groups demonstrated a marked inverse association between serum DHEAS contents and the urine albumin-to-creatinine ratio, suggesting a protective effect of DHEAS against microvascular injury [30]. Although that investigation primarily focused on diabetic nephropathy, the findings imply that the vasoprotective properties of DHEAS may extend to other microvascular beds, including the retinal circulation. In addition, a Chinese study of DR reported substantial negative link between serum DHEA concentrations and DR severity. Given that DHEAS is the sulfated, more stable, and more abundant circulating form of DHEA, reduced DHEAS levels may similarly increase susceptibility to DR [14]. In line with these observations, the current study demonstrated that serum DHEAS levels remained inversely linked with DR risk in men even after adjusting comprehensively for possible confounders, thereby reinforcing prior hypotheses regarding its protective role.

DR is among the most common and severe microvascular diabetes complications and is characterized by a series of pathological alterations, including retinal capillary occlusion, pathological neovascularization, and macular edema [31–33]. Chronic hyperglycemia induces endothelial dysfunction, basement membrane thickening, and increased vascular permeability in retinal capillaries, ultimately leading to impaired perfusion and retinal ischemia [34]. Increasingly, DR is recognized as a neurovascular inflammatory disorder, in which persistent low-grade inflammation contributes substantially to disease initiation and progression [35, 36]. The diabetic retinal microenvironment is marked by sustained inflammatory activation, which promotes microvascular damage and retinal neurodegeneration. Pro-inflammatory TNF-α, IL-6, and IFN-γ are closely involved in this process by inducing endothelial dysfunction, leukocyte adhesion, and increased vascular permeability, thereby facilitating neovascularization and exacerbating DR progression [37–39].

DHEAS possesses potent anti-inflammatory properties, including the ability to suppress the transcription and secretion of pro-inflammatory cytokines. For instance, research examining inflammation and oxidative stress induced by oleic acid in chicken hepatocytes and liver cancer cells revealed that DHEAS blocked NF-κB activation and downregulated the expression of inflammation-related genes [40]. NF-κB is a central transcriptional controller of inflammatory mediators and has been strongly implicated in the inflammatory cascade underlying DR pathogenesis [41]. By preventing NF-κB nuclear translocation, DHEAS reduces the levels of inflammatory cytokines, thereby mitigating inflammatory injury to retinal cells. In addition, DHEAS influences immune cell behavior by modulating macrophage and microglial polarization, suppressing pro-inflammatory phenotypes while promoting anti-inflammatory states, which may help restore retinal microenvironmental homeostasis [42]. In parallel with its direct anti-inflammatory actions, DHEAS also exerts indirect anti-inflammatory effects by alleviating oxidative stress. A study investigating the anti-aging effects of crocin in mice demonstrated that crocin treatment increased serum DHEAS levels, which was accompanied by reduced oxidative stress and attenuated inflammatory responses [43]. Hyperglycemia-induced oxidative stress is a well-established trigger of inflammatory signaling pathways in DR. DHEAS has been shown to scavenge excess reactive oxygen species and reduce oxidative damage, thereby dampening inflammation driven by oxidative stress [40]. Given that DR is fundamentally an inflammation-associated diabetic complication, the combined antioxidant and anti-inflammatory actions of DHEAS may partially explain its inverse association with DR risk in men.

Importantly, DR is not solely a vascular disorder, as neuronal injury and apoptosis within the retina represent critical components of its pathophysiology [44, 45]. DHEAS has been reported to delay neurodegenerative processes by promoting retinal neuronal survival, thereby helping preserve retinal structure and function. Mechanistic studies indicate that DHEAS can activate intracellular anti-apoptotic signaling pathways, enhance neuronal antioxidant defenses, and suppress oxidative and inflammatory damage induced by chronic hyperglycemia [46]. Furthermore, experimental work in male Wistar rats has shown that DHEAS stimulates the expression and secretion of neurotrophic factors such as nerve growth factor (NGF) and brain-derived neurotrophic factor (BDNF) [47]. These factors support neuronal survival, proliferation, and neurite extension, thereby enhancing neural repair and regenerative capacity. Additional evidence suggests that DHEAS may facilitate functional recovery of injured neurons and potentially promote neurogenesis through activation of relevant signaling pathways. Together, these results indicate that the neuroprotective properties of DHEAS may represent an important biological mechanism underlying its association with reduced DR risk in men.

Several limitations of this study exist. First, its cross-sectional nature precludes causal inference. It thus remains unclear whether low DHEAS contributes to the onset of DR or arises as a result of disease progression. This requires further investigation. Second, all participants were recruited from a single hospital inpatient setting, which may limit the generalizability of our findings to broader populations. Hospitalized patients with T2DM typically exhibit more advanced disease, poorer glycemic control, and a higher prevalence of comorbidities compared to community-dwelling individuals. This selection bias may have influenced the observed associations, as the relationships between androgens and DR could vary in severity or direction across the spectrum of diabetes. Third, in the female population, no significant correlation was observed between androgens and DR. This lack of correlation may be attributed to the low statistical power resulting from the relatively small sample size in the female subgroup, as well as the considerable within-group variability introduced by unrecorded menopausal status. These factors may obscure the true association. Fourth, although extensive adjustment for potential confounders was performed, residual confounding from factors such as dietary habits, physical activity, and detailed medication use cannot be excluded. Finally, the relatively modest sample size underscores the need for larger, multicenter studies to confirm and extend these observations.

## Conclusion

In conclusion, the present findings suggest that serum DHEAS may serve as a complementary biomarker for assessing DR risk in male patients with T2DM. Incorporating DHEAS into risk assessment models could support high-risk patient identification beyond traditional clinical factors, thereby facilitating more refined risk stratification. Although androgen-based interventions are not currently recommended for DR prevention, this study provides a theoretical framework for future research exploring targeted strategies in male patients with low DHEAS levels.

## Data Availability

The dataset generated and/or analysed during the current study is available from the corresponding author on reasonable request.
